# Evolution and lessons from an integrated service delivery network in North West Syria

**DOI:** 10.1186/s13031-023-00510-3

**Published:** 2023-03-24

**Authors:** Sophie Witter, Karin Diaconu, Ibrahim Bou-Orm, Zeina Jamal, Zubin Cyrus Shroff, Abdulbaki Mahmoud, Mahmoud Daher, Vinod Varma

**Affiliations:** 1 Institute for Global Health and Development, Queen Margaret University, Edinburgh, UK; 2 ReBUILD for Resilience research consortium, Liverpool, UK; 3 Alliance for Health Policy and Systems Research, World Health Organization, Geneva, Switzerland; 4 World Health Organisation Field Presence in Gaziantep, Gaziantep, Turkey

**Keywords:** Syria, Health service delivery, Essential service package, Provider network, Evaluation, Complex emergencies, Resilience

## Abstract

**Background:**

Northwest Syria (NWS) is a complex and extremely fragile operating environment, with more than 2.8 million people needing humanitarian assistance. To support a common standard of care delivery and enable coordination among the multiple providers in NWS, WHO developed an Essential Health Services package (EHSP) in 2016-17 and subsequently supported a facility network model to deliver the EHSP. This article provides an evaluation of the network to date, aiming to inform further development of the network and draw wider lessons for application of similar approaches in complex emergency settings.

**Methods:**

This mixed method study included document review, participatory, qualitative and quantitative data, gathered in the first half of 2021. Participatory data came from two group model building workshops with 21 funders and implementers. Semi-structured interviews with 81 funders, health professionals and community members were also conducted. Analyses of the workshops and interviews was inductive, however a deductive approach was used for synthesising insights across this and the document review. The final component was a survey of health providers (59 health care professionals) and service users (233 pregnant women and 214 persons living with NCDs) across network and other comparable facilities, analysed using routine descriptive and inferential statistics. Findings across all methods were triangulated.

**Results:**

The study finds that the network and its accompanying essential service package were relevant to the dynamic and challenging context, with high but shifting population needs and multiple uncoordinated providers. Judged in relation to its original goals of comprehensive, coordinated services, equitable access and efficient service delivery, the data indicate that gains have been made in all three areas through the network, although attribution is challenging, given the complex environment. The context remains challenging, with shifting boundaries and populations displaced by conflict, difficulties in retaining staff, the need to import medicines and supplies across borders, and governance gaps.

**Conclusion:**

This study adds to a very limited literature on coordinated network approaches used to raise care quality and improve referrals and efficiency in a complex emergency setting. Although areas of ongoing challenge, including for sustainability, are noted, the network demonstrated some resilience strategies and can provide lessons for other similar contexts.

**Supplementary Information:**

The online version contains supplementary material available at 10.1186/s13031-023-00510-3.

## Background

After 11 years of war, Syria crisis is still characterized by huge suffering and humanitarian needs. Some 6.9 million Syrians are internally displaced, and another 5.6 million are refugees abroad. The situation in northwest Syria (NWS) remains a complex and extremely fragile operating environment. NWS is home to 4.4 million residents as of April 2022, with roughly two-thirds being internally displaced persons (IDPs), many of whom have fled multiple times since the start of the conflict. Of these, 1.7 million are living in tent encampments that are prone to flooding and are exposed to frigid temperatures during the winter. The Humanitarian Needs Overview for NWS for 2022 [1] states that an estimated 3.1 million people are food insecure and it is estimated that a similar number require assistance with health services. The context is further complicated by the growing economic crisis related to the rapid depreciation of the Syrian pound.

Among the 12 million people in need of health services in Syria, 2.7 million are in the north-west. Persons living with disabilities are estimated as 15% of the population (3.07 million people), as well as those with functional difficulties whom require specialized services and access considerations. In NWS, up to 27% of the population is estimated to be impacted by a disability, so over 1 million people could be affected [2]. Relatedly, persons living in areas with high levels of explosive ordnance contamination are vulnerable to traumatic and complex injury and subsequent long-term impairments.

Provision of effective and continuous health care services under such conditions is particularly challenging. Despite populations largely concentrating in urbanised areas, insecurity and shifting lines of authority mean that access to health facilities is compromised both for those seeking as well as delivering care [3]. Further, governmental and public capacity for coordination of service delivery is quasi-non-existent, and while existing health facilities in the areas may continue operating, they do so irregularly, under the auspices of different non-governmental organisations (NGOs) which are supported through humanitarian assistance [4]. Informal private providers also operate here, often with little coordination among themselves and mechanisms of referral to other facilities which may be better equipped and capacitated to address patient needs [5, 6].

While there are benefits in NGOs, relief and humanitarian agencies stepping in to support the available health facilities in maintaining care access, two challenges became evident over time. First, many donors and actors were not aware of how protracted the nature of the Syrian crisis would be and insisted on delivery and implementation of services as designed for short term crisis response. While some of these services were obviously appropriate, the needs of those with chronic conditions or more complex care needs – which present a significant proportion of the Syrian population – could not be covered under such models [7, 8]. Second, delivering care in close quarters via a set of existing facilities lacking coordination presents its own set of unique challenges. For example, all facilities relied on the same health care personnel to deliver care, creating competition and over time exacerbating staff turnover at facility level. Services were likely to be duplicated but also offered to different care standards, as facilities were following guidance of their respective donors. Referral among facilities, despite being necessary and driven by both stock outs and high turnover of the scarce and finite human resource in the area, was rare and under-utilized [6].

WHO has been providing humanitarian and technical assistance to the population of NW Syria through cross-border operations since the UN Security Council Resolution (UNSCR) 2165 was signed in 2014. The WHO Gaziantep Field Presence, through its implementing partners (IPs), which are local NGOs, provides health care, medicine and medical supplies, and public health services to NWS.

To support a common standard of care delivery and enable coordination among the multiple providers in NWS, the WHO Field Presence in Gaziantep – as coordinator of the humanitarian health cluster^1^ in the region – developed an Essential Health Services Package (EHSP) in 2016 [9] and subsequently supported a facility network to deliver the EHSP [10]. The network aimed to integrate the operations of health facilities and ensure the coordinated and comprehensive delivery of care services, with a specific focus on referrals, with the ultimate aim of increasing access to health services for displaced and host populations in the region and ensuring efficient service delivery. To be able to implement the network, WHO coordinated with various donors to support harmonisation of services and standards, as well as to coordinate service inputs (e.g. ensuring access to relevant infrastructure, standardised remuneration for health professionals) [11].

This article examines how the Harim network, an integrated health service delivery network in the Harim District of NWS, was developed and how it evolved over its four years of operation (2017–2021). We provide preliminary evidence on the networks’ effectiveness, discuss opportunities for strengthening, with particular focus on improving outputs and outcomes related to reproductive, maternal and child health and chronic non-communicable diseases. As we enter an era of protracted conflict that increasingly results in the displacement of persons with complex care needs towards urban areas with substantive existing health infrastructure and capacity, the Harim model and lessons learned from its implementation may provide valuable insights for other fragile settings and complex operating environments. In our discussion, we present reflections on how the network may have demonstrated or contributed to resilience capacities in this turbulent region.

## Methods

This was a mixed method study. Document review, group model building (GMB) workshops and key informant interviews were used to understand how and why the Harim network was called into being and how it evolved over time; these also helped identify opportunities for network strengthening. Analysis of routine data, alongside surveys with health providers and patients, further helped establish whether and how the network succeeded in meeting its goals regarding effective service coverage and delivery.

### Document review

The following documentation was reviewed and key themes relating to network evolution and operations narratively synthesised:

- Terms of reference and reports corresponding to the establishment of the network and its renewal over the period October 2017 – February 2021;
- The Essential Health Service Package as initially developed in 2016;
- Quality and gap analyses reports which reported on previous assessments of the network conducted in 2017 and further in 2019–2020;
- A report on the Essential Health Service Package review, commissioned in 2020 by the Health Cluster and conducted by a mixed team, including WHO and local NGOs under the Health Cluster, considering the network and its future consolidation;
- Service costings within the network, carried out in 2017 and 2020.

### Routine data analysis

Complementing the document review, routine data captured by the health cluster across the period 2015–2020 were also reviewed. Specifically, we reviewed routine indicator data as captured in:

- **4Ws (Who does what, where and when)**: these reporting tools summarise who provides what services where and when on a monthly basis;
- **The Health Resources and Services Availability Monitoring System (HeRAMS)**: these capture data relevant to service delivery, such as number of outpatient consultations delivered or services accessed.

While we intended initially to present trends in service delivery to support our understanding of the effectiveness of the network over the years, the datasets did not allow such analysis due to the high number of missing values. We have however presented aggregate figures as reported in periodic reports.

### Group model building workshops

We conducted two GMB workshops in April 2021 to explore the perception of different stakeholder groups in relation to how the network had evolved over time and to identify opportunities for strengthening of the network. Workshops were conducted remotely, online via Microsoft (MS) Teams, over a period of 3 h. This methodology was chosen in order to bring participants together and directly discuss and address differences in perception (e.g. on the reason for why the network was convened, the network’s operations and effectiveness).

#### Sampling and participants

Sampling for the workshops was purposive and convenience based. One workshop targeted donor representatives and WHO staff involved in financing and overseeing the functions of the network (6 participants of a potential 8). The second workshop targeted health professionals and mangers active in the network, as well as network coordinating staff and representatives of the local health directorate (14 participants). Workshops were conducted separately for these groups to ensure that participants were able to converse freely and not be constrained by power-relations (e.g. local staff as opposed to donors); to ensure that local staff were fully comfortable, the latter workshop was conducted in Arabic.

All workshop participants were contacted by the WHO Field Presence in Gaziantep and the study team and sent an information sheet about the study. Participants were assured that views would be kept confidential to the workshop and were also notified that they may be contacted for follow-up individual key informant interviews. Participation in workshops was viewed as confirmation of consent to take part in the study.

#### Workshop activities

Both workshops began by first introducing the study and the study team. The first workshop was conducted in English, and facilitated by members of the research team. The second workshop was conducted in Arabic, and facilitated by the research team in partnership with WHO. After introducing the study and the study team, each workshop proceeded to focus on a similar set of activities – detailed in Appendix 1.

#### Analysis and presentation of findings

Workshops resulted in the development and collection of diverse materials (e.g. drawings of perceived trends relating to how the network evolved over time, audio recordings and casual loop diagrams (CLD) summarising perceptions of participants on the network, including its functions, capacities and ability to meet community and patient needs).

Analyses of these materials proceeded iteratively. Each of the facilitators listened to workshop recordings, refining the materials collected – especially the CLD – as relevant and producing a summary of key insights from each workshop. Key themes corresponding to findings from each workshop were then discussed by the research team and triangulated against findings from the other methods.

### Key informant interviews (KIIs)

To complement the findings of the workshops and probe more deeply for information, and also to capture views of stakeholders not directly involved with the network such as patients and community members, individual semi-structured interviews were also conducted.

#### Participants and data collection

Table 1 outlines characteristics of the 83 key informants interviewed. We targeted participation of persons who had more than one year of experience within their role and who institutionally held experience of the network and its function. Community members and patients were selected using purposive and convenience sampling. They were all adults ≥ 18 years and residing within the network’s catchment area.

**Table 1:** KII participants summary

Participant category	Participant details	Number
Network funders and donors*	Donor representatives	7
Health and network professionals both within and outside network^¥^	Network coordinators and administrators	5
	Health facility managers and health professionals active outside the network’s activities	24
	Health facility managers and health professionals active in the networks’ activities	19
Community health workers^¥^	Active in catchment areas of targeted facilities	6
	Active outside catchment areas of targeted facilities	10
Community leaders and patients^¥^	local leaders such as: teachers, social workers among others. Young mothers, persons with self-reported diagnoses of diabetes and hypertension	12

* *Interviews conducted remotely by research team*

^¥^ *Interviews conducted in person by field surveyors trained by research team*

The study was explained to all interview participants, and oral consent obtained prior to commencement of interviews. Interviews were conducted in English and Arabic and lasted for 30 min on average. They were audio-recorded upon the participant’s consent.

#### Analysis

All audio-recordings of interviews conducted with donors and WHO staff were transcribed verbatim and analysed deductively. For interviews where recordings were not available, these were summarised in note form and notes formed the basis of analysis. Analysis focused on identifying themes of relevance around: perceptions of why and how the network emerged, perceptions of the vision and performance of the Harim network currently, challenges and opportunities for network strengthening. To ensure that different views were accurately captured, data was summarized separately for each category of respondent. Interview data from community members and patients identified themes around: barriers/facilitators to health service access, the main health services utilised, and satisfaction around current services and ways for improvement, marking any changes in health seeking behaviours and available health services since 2016 onwards.

### Surveys

The final component was a cross-sectional survey of health providers (59 health care professionals from facilities inside and outside the network) and service users (233 pregnant women and 214 persons living with non-communicable diseases (NCDs)) to assess perceptions of the capacity, efficacy and effectiveness of the primary care network, and patient satisfaction.

#### Target population and eligibility criteria

Surveys were carried out in purposively selected facilities both inside and outside the network. Selection was based on comparability considerations: as we intended to compare key indicators among patients accessing care in the network to those outside, we purposively selected facilities that were geographically serving distinct populations and were of similar size and make-up, both inside and outside the network. We carried out surveys in three facilities within the network (including one hospital, and two primary healthcare centres (PHCCs)), and five outside (including one hospital, two PHCCs and two mobile clinics).

#### Sampling

Within these facilities, samples for health professionals were purposive and convenience based in that only available staff with sufficient time were able to participate. Appendix 2 sets out the required sample size to assess differences in key services delivered in facilities within the network and those outside. For the patients, we did not segregate host population and IDPs but surveyed in areas with high numbers of IDPs, reflected in the final proportion of respondents (78% overall were IDPs – see Appendix 3).

#### Data collection

Trained data collectors approached potential participants at the health facilities sampled, offering each participant an oral and written explanation of the study and further securing their oral consent for participation. For community members, persons were approached both at health facilities and also in the catchment areas of health facilities. For the latter, persons were asked where they accessed care most frequently to determine eligibility for taking part in the survey. All surveys were carried out in Arabic and were pilot-tested prior to roll-out in March 2021.

For health professionals and facility managers, surveys focused on questioning perceptions surrounding health systems functions, service delivery, community engagement, perceptions of current effectiveness of service delivery around the tracer conditions, capacity to provide integrated services for tracer conditions, and experiences of working with community-based health workers.

For patients, surveys focused on barriers/facilitators to health service access, delivery and utilization of services in line with the essential health service package, perceptions of quality and comprehensiveness of care delivery, and patient satisfaction with services.

Most variables were constructed in the form of 5-point Likert scales measuring the extent of agreement (or disagreement) with specific statements or rating constructs like satisfaction domains.

#### Analysis

We carried out complete case descriptive analyses of the data, including univariate and bivariate analyses. We present analyses by participant group (noting that the sample is powered only for the patient groups), distinguishing as appropriate between pregnant women and people living with NCDs. Where relevant, categories with small numbers were merged to allow for more meaningful comparison and to avoid zero or small values in categories and ensure the validity of used statistical tests. Categories were only merged if they were in the same direction of agreement and along with ‘neutral’ options if appropriate. In this case, the p-value would indicate whether there is a statistical difference in the magnitude of agreement or disagreement. We mainly used the following statistical tests to test the association between variables from the previously mentioned topics and the affiliation of facilities to the network (as a binary variable): t-test: in case of a continuous variable such as satisfaction score or participant age; and Chi-square test in case of a categorical variable with an appropriate distribution of values between categories. An association is deemed significant if the p-value is less than 0.05. We used IBM SPSS v.23.

### Ethics and quality assurance

#### Ethical approval

was obtained from Queen Margaret University in January 2021. Local ethical approval was not possible in the disrupted circumstances but local partners were consulted during the design stage and to ensure safe and ethical data collection. All data was treated confidentially and stored on password protected computers accessible only by members of the research team. An information sheet / oral consent form describing the aims of the study using simple language was given to participants who were allowed enough time to ask questions prior to agreeing to participate. During data collection, the autonomy and individual privacy of the participants, and their confidentiality of information was respected. Participants were allowed to skip any question and withdraw from any of the data collection methods at any time.

### Limitations

Study limitations include gaps in routine data and that the review was conducted rapidly and remotely, with restricted access due to the geopolitical situation and COVID-19, which limits the depth of information gathered by the team. It is also important to bear in mind the non-random nature of the inclusion of health facilities in relation to survey data and that there may have been contamination from networked facilities to non-networked ones (through the organisations which were managing both).

## Results

We first present findings relating to how and why the network evolved, followed by findings relating to network effectiveness.

### Part 1: Network evolution

As detailed in the introduction, the context in Syria was and remains highly dynamic, with shifting de facto control over territories, large scale population displacement and a large number of health actors, including local and international NGOs, the private sector and development partners [12]. Involvement of these many actors in the delivery of care in relatively close quarters gave rise to specific challenges (see introduction), which WHO, as coordinator of the health cluster emergency response in the region, sought to remedy by developing a common essential health services package, which covered child and reproductive health, communicable and non-communicable diseases, mental health and nutrition (see Box 1, appendices).

The EHSP was defined via a joint exercise between donors, the health cluster, humanitarian partners, implementers and WHO, including by establishing current needs of facilities, and setting out standards of services and service offer sensitive to local needs (KIIs). One aim of the EHSP is to ensure continuity of care, including by ensuring that health practitioners are able to refer patients as relevant for specialised or additional services.

#### Establishment of the network

To ensure effective implementation of the package, and address the coordination and continuity of care challenges in the region, an integrated service delivery network model was proposed. This implied a set of facilities spread across a geographical area agreeing to co-operate in terms of standard operating procedures, staff availability and training, information systems and management, irrespective of service funders. The key components of the network were thus [1] to implement the EHSP (see box 1) and guidelines accompanying it and [2] to promote decentralized and locally sensitive planning, which evolved over phases.

The pilot network of 10 PHC-level facilities started in Saraqib in 2017, and then expanded to include more areas in the south (Maarat al-Numan district) and the west (Idleb district). The area was selected as it was underserved based on data from the HeRAMS, comparatively secure, and in need of improved access to and efficiency in healthcare. Due to military operations in these locations and the waves of displacement towards Harim and other similar areas close to the borderline with Turkey in the second half of 2019, the network was relocated to the Harim area. Selection of the Harim area was influenced by a variety of factors, such as population density, the number of displaced persons, geographical location, the availability of health facilities, and the rapid health facility assessment that WHO contracted a local implementing partner to carry out in 2019 (not published).

Six monthly contracts were established between WHO and the lead NGO implementer (network coordinator), with the latter providing reports which tracked service delivery, quality assessment and challenges, capacity building activities, challenges and actions taken. The first terms of reference for the network listed the goal of the network as increasing access to services (proxied by outpatient consultations) by 30% [13].

#### Evolution of the network

**Fig. 1:**
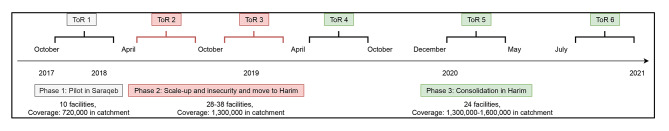
Evolution of the Harim integrated service network, phases 1–6, 2017-21

An overview of network evolution from 2017 to 2021 is provided in Fig. 1. The network, in terms of participating facilities (mobile units, PHCCs, comprehensive PHCCs, with some PHCCs based in hospital sites), has shifted considerably over the period, largely driven by the shifting front of the war, with the population covered by the network growing from 180,000 to 1.6 million, and participating facilities increasing from 10 to 38. Some continued overlap of services was noted by Key-informants (KIs). The goal of increasing service coverage (30% increase in outpatient visits, later expanded with goals for children and NCDs) was met. A description of network evolution by health system building block follows.

##### Health system governance

WHO has played a lead role, coordinating the project under the umbrella of the Health Cluster, and providing technical support, capacity building, quality assurance, monitoring and evaluation. The programme was implemented by a local NGO (acting as the network coordinator), working with a variety of providers and facilities, and reporting to WHO, while also coordinating with the health directorates of the relevant districts. However, the governance structures have been strengthened since 2020 with the development of a ‘network management team’ involving WHO, the health directorates and relevant international non-governmental organisations (iNGOs) at field office level in Turkey, while at the operational level, a coordinating group of network coordinators, implementing NGOs and facility managers has been established. This was in response to the shifting authorities on the ground in north west Syria. There has been greater recognition of the need to build local management capacity (in NGOs and health directorates) to assure services over the longer term.

##### Health financing

Financial support has come from a range of donors over the years (Japan, the Foreign, Commonwealth & Development Office (FCDO) UK, the Office of U.S. Foreign Disaster Assistance (OFDA), and the European Civil Protection and Humanitarian Aid Operations (ECHO)), translating into varying amounts per capita for the covered population, which is hard to assess in relation to adequacy, given the other sources which NGO facilities draw on. Facility budgets are based on gaps in key inputs, and funds are disbursed to the network coordinator, which further subcontracts NGOs. Performance-based payments have not been possible given the dynamic security situation, the fragmented financing sources and limited verification and assurance capacities. Services were intended to be free to users but data to verify the extent of household out of pocket payments has been lacking (EHSP review 2020) [14].

##### Human resources for health

Staffing has been the main expense and considerable effort has also been put into training, however, staff mobility and the competitive (and unregulated) labour market in the areas continue to put a strain on the availability of staff with sufficient competences. In particular, the coordination, referral and data management roles have been combined in many facilities into one overloaded role, according to our interviews.

##### Infrastructure, medicines and equipment

WHO has directly procured and supplied many of the essential drugs, supplies and equipment needed by the network, and continues to do so, although more recently it engaged in the development of a coordinated procurement and distribution system for all the providers participating in the network, led by the network coordinator NGO.

##### Health information systems

There has been a consistent challenge to ensure reporting according to a unified health information system, but since 2018, work has been ongoing to introduce the Digital Health Information System 2 (DHIS2), with some progress in 2020-21 when DHIS2 reporting was made mandatory for the network and some of the constraining factors (such as the need for training and providing dedicated staff) addressed. Some challenges remain however, such as fragmented donor reporting requirements which have led to heavy burdens for facilities in case-based reporting via DHIS2.

##### Service delivery

The content of the EHSP has remained broadly constant over the period, covering six main intervention areas, reflecting essential public health goals. A consistent emphasis has been placed on improving referrals between facilities through sharing maps of services, providing referral protocols and delivering training in their use. Elements such as community outreach and advocacy gained in importance in later phases, recognising their importance, and since 2020 triaging and management of COVID cases was added (KIIs).

KIs noted that the integrated network model was ambitious, aiming to provide more efficient but high-quality coverage to host and displaced populations in a very difficult operating environment. However, stakeholders confirmed the relevance of the network model, especially given the limited resources available for care, the high burden of disease and the prevalence of chronic illnesses such as NCDs and mental health which require greater continuity of care.

**Fig. 2:**
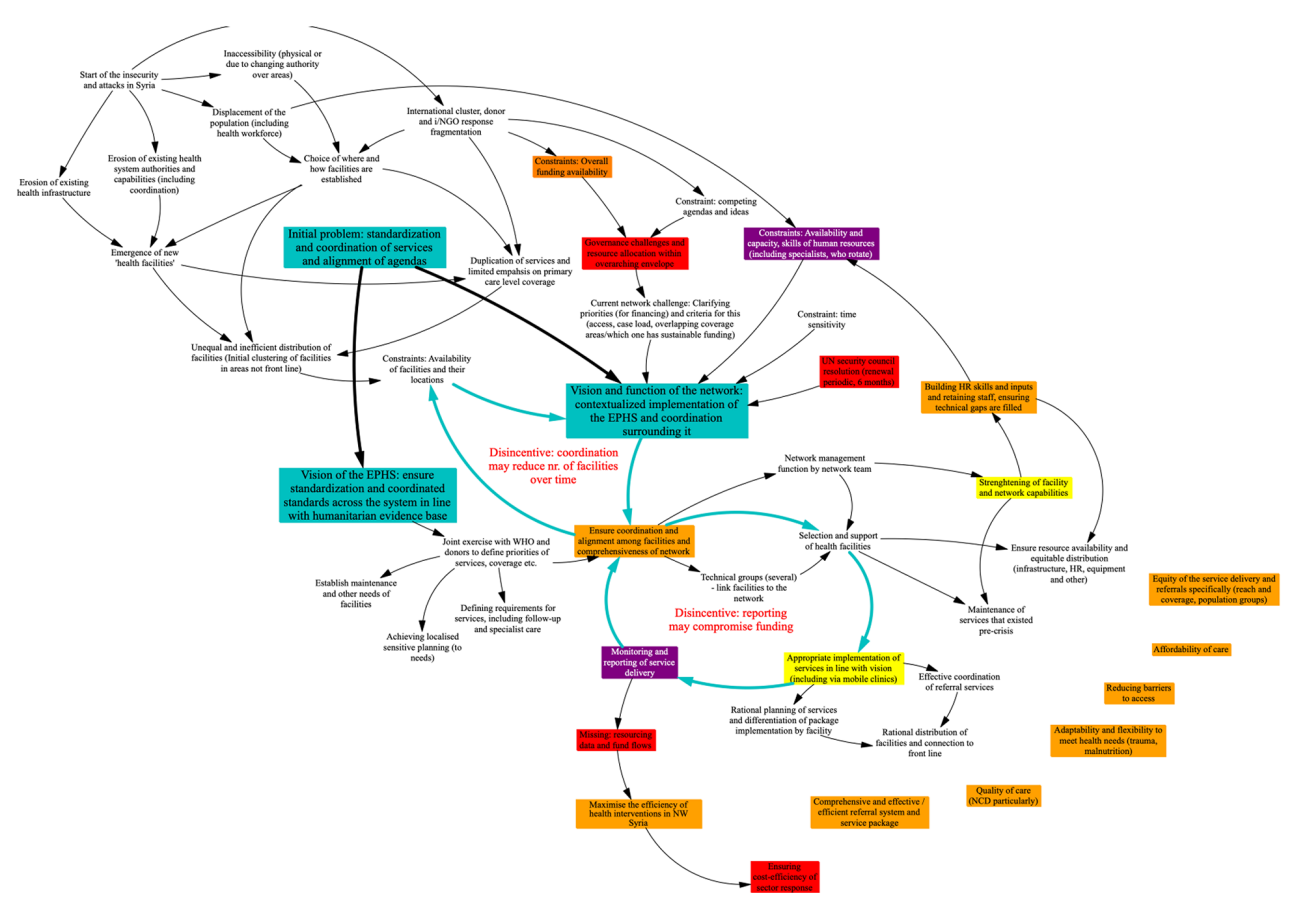
Donor GMB model

Informants discussed the risks which the network tried to address and noted lack of coverage and inefficient coverage (with duplication and overuse of hospital services for conditions which could be managed at primary level) (Fig. 2). In the view of partner KIs, the network served as a vehicle for aligning governance and management, functioning like a mini health system which could support the implementation of the EHSP.

#### Contextual factors influencing evolution

Clearly the dynamic nature of the conflict in Northwest Syria was a key driver underlying many of the developments in the network, through several channels. These include destruction of health facilities, displacement of people and health staff, disruption of training, increasing health needs in the community, and disruption of the network, for example in terms of attacks on staff and disruption of finance and supplies (GMB workshops).

This insecurity also impacted on the facilities included in each phase, which varied over time, with the network shifting to accommodate the moving conflict. This is likely to have impacted on the ability to train staff and build capacity that was maintained across the network.

The governance context has also been challenging: the withdrawal of the state administration from insurgent areas encouraged self-organisation efforts by former health departments (now health directorates) (KII). According to the EHSP review of 2020, ‘the relative weakness of health governance mechanisms in north-west Syria remains one of the root causes of fragmented health service delivery because there is limited stewardship for making the most out of scarce resources in terms of equitable access to primary care. The ‘Health Directorates’ have gained experience as de-facto decentralized health governance structures but face significant contextual challenges, and lack of funding and support’ [14]. It is however important to recognise the different contexts across areas, as there are multiple de facto authorities with varying roles and reach, as is common in complex emergency settings (KII). The network has focused on building managerial capacity in implementing NGOs and facilities, for example, through its use of service agreements with clear targets, introduction of tools such as patient satisfaction surveys, and needs assessments, capacity building and supportive supervision (KII).

An underlying challenge for the network is the lack of availability of human resources for health in the region and human resource management challenges (EHSP review 2020) [14]. One hospital manager highlighted that they still had to rely on staff (including nurses, midwives and paramedics) who have work experience but lack the relevant academic training, given the disrupted context. This was also true of health coordinators and managers. KI stress that human resource issues are perhaps the most pressing concern in the region, as there is a lack of regulation, lack of norms and uniform salary scales and irregular payments. For example, different NGOs within one area may use different pay scales, creating perverse incentives in terms of overall distribution of the workforce.

*‘We face a problem that the staff is employed elsewhere and therefore there is no coordination of resources in general at the central level and there is a loss of resources as a result of the change. For example, a nurse may be trained on a specific device and due to the expiration of his/her contract or his/her desire to move to get a better salary, he/she may move to a place where his/her training is not relevant’* (HR staff- Outpatient Clinic).

Systems to manage dual practice by health staff are also lacking and vertical programmes operate independent systems for staffing and pay. The vacuum in governance due to weak health authorities has left professional categories and the respective training programs that were present earlier to fossilize, while new healthcare cadres have been promoted by aid agencies. In particular, community-related profiles have been evolving in isolation and working as vertical programs detached from health facilities (community health workers (CHWs) and cadres deployed for immunization and mental health) to meet donor priorities, which has led to increase in fragmentation. There is also no harmonised training needs assessment or delivery.

### Part 2: perceptions on the effectiveness of the Harim network

#### Results from provider perspective

We recruited a total of 59 participants, of which 26 (44%) worked inside the network. Participants were predominantly men (61%) with a mean age of 35 years. Most surveyed participants within the network were health providers (10 out of 26), followed by community health workers (7 out of 26), however, the most reported occupation outside the network was clerk or technician (about 50%), followed by community health workers (9 out of 33).

#### Quality of care

Service providers working in and outside the network were asked to state the extent to which they agreed with several statements indicative of care comprehensiveness, triage and coordination (Table 2). Almost all participants within and outside the network agreed that their facility responds effectively to urgent and acute cases using a triage process and that planning and scheduling of non-acute consultations is done in a timely and effective manner. In terms of comprehensiveness of care, participants within the network had a higher agreement regarding the ability of their facility to deliver comprehensive services compared to those outside the network (p = 0.037). The most common type of medical records within and outside the network was based on both paper and electronic formats. Referral systems to specialist consultations and back to the facility existed in both settings without statistically significant difference. Overall, only confidence in the comprehensiveness of the package on offer by the facility was generally more positively assessed by providers in the network.

**Table 2:** Perceptions of health workers in Idleb on selected service delivery features (N = 59)

Variables	Inside the network (n = 26)	Outside the network (n = 33)	Total (N = 59)	p-value
**Planning and scheduling of consultations for non-acute conditions is done in a timely and effective manner (n, %)**				0.543*
Strongly disagree	0 (0.0)	1 (3.0)	1 (1.7)	
Disagree	1 (3.8)	0 (0.0)	1 (1.7)	
Neither disagree nor agree	1 (3.8)	1 (3.0)	2 (3.4)	
Agree	17 (65.4)	26 (78.8)	43 (72.9)	
Strongly agree	7 (26.9)	5 (15.2)	12 (20.3)	
**The facility responds effectively to urgent or acute cases and has triage processes in place (n, %)**				0.767*
Strongly disagree	0 (0.0)	0 (0.0)	0 (0.0)	
Disagree	1 (3.8)	0 (0.0)	1 (1.7)	
Neither disagree nor agree	0 (0.0)	0 (0.0)	0 (0.0)	
Agree	16 (61.5)	21 (63.6)	37 (62.7)	
Strongly agree	9 (34.6)	12 (36.4)	21 (35.6)	
**The facility is able to provide comprehensive services (n, %)**				0.037*
Strongly disagree	0 (0.0)	0 (0.0)	0 (0.0)	
Disagree	0 (0.0)	1 (3.0)	1 (1.7)	
Neither disagree nor agree	2 (7.7)	9 (27.3)	11 (18.6)	
Agree	18 (69.2)	21 (63.6)	39 (66.1)	
Strongly agree	6 (23.1)	2 (6.1)	8 (13.6)	
**Current medical record system (n, %)**				0.441
Medical records in paper format only	1 (3.8)	0 (0.0)	1 (1.7)	
Medical records both in paper and electronic format	25 (96.2)	33 (100)	58 (98.3)	
Medical records in electronic format only	0 (0.0)	0 (0.0)	0 (0.0)	
**Current referral system (n, %)**				0.108
No referral system present	0 (0.0)	0 (0.0)	0 (0.0)	
Referrals only available for diagnostic or laboratory services	2 (7.7)	4 (12.1)	6 (10.2)	
Referral to higher levels for specialist consultation usually available	12 (46.2)	7 (21.2)	19 (32.2)	
Referral to higher levels for specialist consultation and follow-up from that specialist consultation back to the health facility (counter-referral)	11 (42.3)	22 (66.7)	33 (55.9)	

#### Teamwork, management and job satisfaction

Among the remaining assessed topics in this survey, only a few differences around teamwork, management and job satisfaction were reported (Table 3). Providers in the network had more positive views on coordination of the health care team within the facility and the availability of resources needed to maintain equipment than those outside. However, there were also areas where staff outside the network were significantly more positive, including on satisfaction with recognition (also greater satisfaction with working hours and pay, though this was not significant) and believing that their views would be taken into account by management, which may be linked to higher utilisation of facilities within the network.

**Table 3:** Selected findings from the survey with health providers on teamwork, management and job satisfaction (N = 59)

Variables	Inside the network (n = 26)	Outside the network (n = 33)	Total (N = 59)	p-value
Disagreements in this health care facility are resolved appropriately (i.e. not who is right, but what is best for the patient)				0.041
Strongly disagree	0 (0.0)	0 (0.0)	0 (0.0)	
Disagree	0 (0.0)	0 (0.0)	0 (0.0)	
Neither disagree nor agree	0 (0.0)	2 (6.1)	2 (3.4)	
Agree	16 (61.5)	26 (78.8)	42 (71.2)	
Strongly agree	10 (38.5)	5 (15.2)	15 (25.4)	
**The staff at this health care facility work together as a well-coordinated team**				**0.017**
Strongly disagree	0 (0.0)	0 (0.0)	0 (0.0)	
Disagree	0 (0.0)	0 (0.0)	0 (0.0)	
Neither disagree nor agree	0 (0.0)	0 (0.0)	0 (0.0)	
Agree	10 (38.5)	24 (72.7)	34 (57.6)	
Strongly agree	16 (61.5)	9 (27.3)	25 (42.4)	
**Management supports this facility to maintain equipment and to repair/replace it if is broken**				0.051
Strongly disagree	0 (0.0)	0 (0.0)	0 (0.0)	
Disagree	0 (0.0)	1 (3.3)	1 (2.0)	
Neither disagree nor agree	1 (4.8)	0 (0.0)	1 (2.0)	
Agree	12 (57.1)	25 (83.3)	37 (72.6)	
Strongly agree	8 (38.1)	4 (13.3)	12 (23.5)	
**How satisfied or dissatisfied are you with the recognition you get for good work**				**0.033**
Not at all satisfied	0 (0.0)	0 (0.0)	0 (0.0)	
Not satisfied	4 (15.4)	1 (3.0)	5 (8.5)	
Neither satisfied or dissatisfied	7 (26.9)	2 (6.1)	9 (15.3)	
Satisfied	12 (46.2)	24 (72.7)	36 (61.0)	
Very satisfied	3 (11.5)	6 (18.2)	9 (15.3)	
**My supervisor takes into consideration my views and ideas**				**0.002**
Strongly disagree	2 (10.5)	0 (0.0)	2 (4.3)	
Disagree	1 (5.3)	0 (0.0)	1 (2.1)	
Neither disagree nor agree	1 (5.3)	1 (3.6)	2 (4.3)	
Agree	14 (73.7)	13 (46.4)	27 (57.5)	
Strongly agree	1 (5.3)	14 (50.0)	15 (31.9)	

These findings should be interpreted with caution as facilities outside the network may be affiliated to the same NGOs as those included; thus, benefits of the network (through training and systems, for example) would be diffused across both potentially. Moreover, all facilities are in receipt of some level of support, which we were not able to assess or control for.

#### Qualitative feedback on the network

Interviews with providers suggested broad satisfaction with the network and recognition that it has been adaptive. Staff trainings on the EHSP, circulation of protocols and providing logistic support to health facilities, including providing the needed equipment, medications and referral vehicles, were seen as important network activities. On top of these, the development of a referral system that is well coordinated and communicated, the development of an appointment system, and the increase in trained staff, were mentioned by staff in different types of facilities.

*‘The current EHSP is pretty capable of meeting community needs compared with the previous situation in 2016, of course; the services at the centres have improved substantially’* (CHW – PHCC).

‘*The human cadre working within this network has increased, and the network has gained a good reputation among most organizations, and there is a certain desire to join this system and work within it*’ (Physician-Outpatient Clinic).

*‘With regard to ensuring service access, we have acquired a health map for all the facilities and services that exist in these establishments, in addition to assigning communication officials who organize referral processes in these establishments. Increasing the awareness of beneficiaries on the services in each facility and the services found in the rest of the facilities within the network by distributing brochures so that every patient knows where to go to receive a specific service’ (Field Medical Coordinator)*.

Gap areas highlighted by staff and managers included: overall resourcing being inadequate, lack of emergency transportation, shortfalls in medicines for chronic conditions and gaps in certain medical specialties.

*‘The issue of financial flow remains a large deficit, which the network is not expected to solve, but at least it will mitigate its bad effects on the beneficiaries*” (Network Coordinator – PHCC).

The need for integration of strong community outreach and actions to address mental health needs, including stigma in using services, was also highlighted.

Interviews with staff from non-networked facilities found some similar themes, but less consistency in how referrals were being managed, compared to the network staff. There is likely some spill over of the model as the same NGOs are managing facilities within and outside the network. Managers reported challenges, including lack of security around the facilities and attacks on staff, work pressures, staff moving to other areas, lack of transport for staff and for patient referral, lack of medicines, infrastructure challenges and low health literacy in the community.

Participants in the network reported some areas of improved efficiency. For example, health coordinators, facility managers and providers mentioned in the GMB workshop that the development/implementation of EHSP had strengthened the network by: [1] the standardization of protocols and guidelines for services, staffing, infrastructure, medicines and (after 2019) for operational planning; [2] shifting the attention and priorities of donors to support a new model of service delivery away from funding facilities in a siloed manner; [3] better governance arrangements. The latter was linked by the participants to increased coordination between facilities and the efficient use of available resources. As a result, triage and referral mechanisms were established and led to decreasing pressure on hospitals, retaining patients within the network and ensuring follow-up, and improving quality of services in general. Those mechanisms contributed to the reduction of duplication of services, which fed back into more efficient use of available resources.

### Results from patient perspectives

In this survey, we recruited 447 persons with 177 persons accessing services at facilities within the Harim network, 190 participants accessing services outside the network and 80 from mobile clinics outside the network. The sample included 233 pregnant women and 214 people living with NCDs (hypertension and diabetes). Mean age was about 40 years (SD = 14.7). Most respondents were women including among the NCD subgroup, which included 132 women (62% of this subgroup). More characteristics of our sample are available in appendix 3 – Table 1.

Overall, the patient survey (Appendix 3; Tables 2 and 3) presented an encouraging picture. On access to care, significant differences were found between persons accessing networked facilities, compared to those without, with those using facilities in the network reporting lower physical, financial and cultural barriers. In relation to financial barriers (Appendix 3; Table 2), about 37% of participants within the network reported facing financial barriers often or every time they sought care, compared to 51% among those outside the network (p-value < 0.001). Looked at in relation to the health condition sub-groups (results not shown), the difference in reported security and financial barriers was significant for both groups, but physical barriers were only significant for NCD patients, while psychological and cultural barriers were only significant for pregnant women.

Relating to experiences with facilities and patient satisfaction, most users went to their facility as a first choice, with higher percentages among people living with NCDs within the network, and were confident to be seen that day. In relation to satisfaction with staff, results were generally positive, with staff at network facilities being reported to be more likely to understand the user’s problem for NCD patients. Follow up on referrals was also reported more frequently for network facility users (general and for both sub-groups). Pregnant women reported greater privacy in the network facility group, for example, and NCD patients reported higher staff skills, but lower involvement of themselves in decision-making in the network facilities. On facility quality of care, answers were mixed, with NCD patients showing more variation in scores in general (perhaps reflecting their chronic engagement). For CHWs, there was general high confidence, but less so among NCD patients in the network group compared with the non-networked group. Appendix 3 – Table 3a and 3b show disaggregated data by study sub-groups (of pregnant women and people living with NCD).

Our survey also measured health outputs among the 2 sub-groups. In terms of NCD conditions (hypertension (HTN) and diabetes mellitus (DM)), there was no difference in duration of condition within/outside the network, or in number of reported consultations in the last year. Based on the last reported HTN and DM readings, we found very low levels of disease control in all facilities - only 17% of those who reported their HTN readings and 11% of those who reported their DM readings had controlled diseases. However, screening for complications was significantly higher in the network group - about 31% of people with HTN and 37% of those with DM within the network had received an eye examination in the last year before the survey, which was around double the rate in the non-network group. About half of all respondents had referrals for their NCD and most of them (75%) were either satisfied or very satisfied with those referrals. No difference was observed between facilities within and outside the network. However, as reported earlier, people within the network reported better follow-up on referrals from their providers. Table 4 presents all NCD-related health outputs measured in our survey.

**Table 4:** Health outputs among people living with NCD in Idleb (N = 214)

Variables	Within the network	Outside the network	Mobile Clinics	Total	p-value*
Hypertension-related variables					
**Date of diagnosis – HTN (n = 160)**					0.738
Less than one year ago (n,%)	11 (18.3)	16 (22.2)	6 (21.4)	33 (20.6)	
One year ago or more (n,%)	49 (81.7)	56 (77.8)	22 (78.6)	127 (79.4)	
Years (mean, SD)	5.40 (4.63)	5.64 (3.98)	6.18 (5.80)	5.64 (4.56)	0.777
**Number of consultations for HTN** (mean score ± SD)	5.40 (4.38)	5.92 (3.89)	5.96 (2.85)	5.73 (3.9)	0.474
**Ability to assess blood pressure (BP) in the last 3 months (n,%)**	54 (90.0)	59 (81.9)	28 (100)	141 (88.1)	0.287
**Able to recall and report the last reading (n,%)**	27 (50.0)	41 (69.5)	18 (64.3)	86 (61.0)	0.055
**Systolic Blood Pressure (SBP) in mmHg (mean, SD)**	153 (23)	152 (21)	139 (20)	149 (23)	0.824
**Diastolic Blood Pressure (DBP) in mmHg (mean, SD)**	89 (9)	91 (16)	86 (10)	89 (13)	0.821
Disease control based on the last reading (n,%)**	4 (14.8)	6 (14.6)	5 (27.8)	15 (17.4)	0.999
**Self-assessment of HTN control level**					0.827***
Not at all controlled	*1 (1.7)*	*3 (4.2)*	*1 (3.6)*	*5 (3.1)*	
Not controlled	*13 (21.7)*	*15 (20.8)*	*7 (25.0)*	*35 (21.9)*	
Somehow controlled	26 (43.3)	27 (37.5)	10 (35.7)	63 (39.4)	
Controlled	*20 (33.3)*	*26 (36.1)*	*10 (35.7)*	*56 (35.0)*	
Well controlled	*0 (0.0)*	*1 (1.4)*	*0 (0.0)*	*1 (0.6)*	
**Eye examination – HTN (n,%)**	18 (31.0)	11 (15.7)	10 (40.0)	39 (25.5)	**0.039**
**Diabetes-related variables**					
**Date of diagnosis – DM (n = 117)**					0.390
Less than one year (n,%)	6 (11.3)	9 (19.6)	2 (11.1)	17 (14.5)	
One year or more	47 (88.7)	37 (80.4)	16 (88.9)	100 (85.5)	
Years (mean, SD)	5.95 (6.37)	5.30 (4.05)	3.87 (3.20)	5.37 (5.18)	0.589
**Number of consultations for DM** (mean score ± SD)	6.10 (7.09)	6.80 (3.93)	7.28 (3.71)	6.56 (5.53)	0.549
**Ability to assess your blood sugar in the last 3 months (n,%)**	35 (66.0)	31 (67.4)	14 (77.8)	80 (68.4)	0.999
**Reported the last reading (n,%)**	35 (66.0)	31 (67.4)	14 (77.8)	80 (68.4)	0.999
**Fasting blood sugar (mean, SD)**					
**Disease control based on the last reading of FBS (n,%)**	2 (5.7)	4 (12.9)	3 (21.4)	9 (11.3)	0.408FE
**Self-reported disease control**					0.118***
Not at all controlled	*4 (7.5)*	*3 (6.5)*	*1 (5.6)*	*8 (6.8)*	
Not controlled	*4 (7.5)*	*8 (17.4)*	*5 (27.8)*	*17 (14.5)*	
Somehow controlled	25 (47.2)	26 (56.5)	8 (44.4)	59 (50.4)	
Controlled	*18 (34.0)*	*9 (19.6)*	*4 (22.2)*	*31 (26.5)*	
Well controlled	*2 (3.8)*	*0 (0.0)*	*0 (0.0)*	*2 (1.7)*	
**Eye examination – DM (n,%)**	19 (37.3)	7 (15.6)	8 (44.4)	34 (29.8)	**0.031**
**Both NCD**					
**Referral for NCD (n, %)**	45 (51.7)	52 (50.5)	22 (52.4)	119 (51.3)	0.980
**Patient experience with the referral for NCD (n,%)**					0.973***
Not at all satisfied	0 (0.0)	0 (0.0)	2 (8.0)	2 (1.7)	
Not satisfied	1 (2.4)	1 (1.9)	6 (24.0)	8 (6.7)	
Neither satisfied nor dissatisfied	7 (17.1)	10 (18.9)	3 (12.0)	20 (16.8)	
Satisfied	28 (68.3)	35 (66.0)	13 (53.0)	76 (63.9)	
Very satisfied	5 (12.2)	7 (13.2)	1 (4.0)	13 (10.9)	
**Reported health status (n,%)**					0.459***
Very bad	1 (1.1)	2 (2.4)	1 (2.6)	4 (1.9)	
Bad	20 (22.2)	18 (21.2)	8 (20.5)	46 (21.5)	
Fair	34 (37.8)	39 (45.9)	17 (43.6)	90 (42.1)	
Good	32 (35.6)	26 (30.6)	13 (33.3)	71 (33.2)	
Very good	3 (3.3)	0 (0.0)	0 (0.0)	3 (1.4)	

* comparing the 2 following categories: within the network (n = 87) and outside the network (n = 105)

** SBP < 140 and/or DBP < 90

*** categories with small values were combined to abide by the rules of used statistical tests.

Pregnancy-related service delivery showed major differences depending on the affiliation of facilities. Pregnant women within the network reported higher uptake of tests during the first trimester, compared to those outside the network. For instance, the percentages of blood tests, glucose test and urine tests within the network were 71%, 39% and 90% respectively – compared to 43% (p < 0.001), 25% (p = 0.037) and 47% (p < 0.001) outside the network. Moreover, a higher percentage of pregnant women within the network reported having a physical examination at each visit (53% compared to 19% outside the network – p < 0.001). The remaining outcomes did not differ between facilities within and outside the network, but were high in both settings: 97% of surveyed pregnant women received 2 ultrasounds in the first and second trimester; 96% received or had been prescribed folic acid and about 80% received counselling on danger signs of pregnancy.

When asked about referrals, one third of all pregnant women in this survey reported at least one referral during the course of their pregnancy and the vast majority of them (89%) were either satisfied or very satisfied with the referral experience. No difference was observed between respondents by the affiliation of facilities. Despite a high level of outputs within the network that exceeded those outside the network for some services, the number of consultations for pregnancy were smaller within the network (mean = 2.6 compared to 4.2 outside the network – p < 0.001), suggesting better efficiency of services – especially as our analysis showed no statistical difference between the distribution of pregnancy trimesters among pregnant women within and outside the network. Table 5 presents all pregnancy-related health outputs measured in our survey.

**Table 5:** Health outputs among pregnant women in Idleb (N = 233)

Variables	Within the network (n = 87)	Outside the network (n = 105)	Mobile Clinics (n = 41)	Total (N = 233)	p-value*
**Trimester** (n,%)					0.750
Second	53 (60.9)	61 (58.1)	33 (80.5)	147 (63.1)	
Third	34 (39.1)	43 (41.0)	8 (19.5)	85 (36.5)	
**Number of consultations** (mean score ± SD)	2.6 (1.4)	4.2 (1.6)	2.9 (1.2)	3.4 (1.6)	**< 0.001**
**Blood tests during the first trimester (n,%)**	62 (71.3)	45 (43.3)	13 (31.7)	120 (51.7)	**< 0.001**
**Glucose test during the first trimester (n,%)**	34 (39.1)	26 (25.0)	5 (12.2)	65 (28.0)	**0.037**
**Urine test during the first trimester (n,%)**	78 (89.7)	49 (47.1)	27 (65.9)	154 (66.4)	**< 0.001**
**Ultrasound during 1st trimester (n,%)**	81 (93.1)	104 (100)	39 (95.1)	224 (96.6)	**0.003**
**Ultrasound during 2nd trimester (n,%)**	86 (98.9)	103 (99.0)	41 (100)	230 (99.1)	0.999
**Blood pressure taken at each visit (n,%)**	40 (46.0)	41 (39.4)	18 (43.9)	99 (42.7)	0.444
**Physical Examination at each visit (n,%)**	46 (52.9)	20 (19.2)	10 (24.4)	76 (32.8)	**< 0.001**
**Counselling on danger signs of pregnancy (n,%)**	62 (71.3)	85 (81.7)	36 (87.8)	183 (78.9)	0.124
**Folic acid prescription or delivery (n,%)**	84 (96.6)	98 (94.2)	41 (100)	223 (96.1)	0.513
**Referral (n, %)**	45 (33.1)	64 (39.8)	18 (26.9)	127 (34.9)	0.286
**Patient experience with the referral**					0.601**
Not at all satisfied	0 (0.0)	0 (0.0)	0 (0.0)	0 (0.0)	
Not satisfied	2 (4.4)	2 (3.1)	0 (0.0)	4 (3.1)	
Neither satisfied nor dissatisfied	4 (8.9)	6 (9.4)	0 (0.0)	10 (7.9)	
Satisfied	32 (71.1)	50 (78.1)	14 (77.8)	96 (75.6)	
Very satisfied	7 (15.6)	6 (9.4)	4 (22.2)	17 (13.4)	
**Reported health status (n,%)**					0.708
Very bad	5 (2.8)	8 (4.2)	1 (1.3)	14 (3.1)	
Bad	37 (20.9)	34 (17.9)	13 (16.3)	84 (18.8)	
Fair	62 (35.0)	74 (38.9)	36 (45.0)	172 (38.5)	
Good	64 (36.2)	68 (35.8)	27 (33.8)	159 (35.6)	
Very good	9 (5.1)	6 (3.2)	3 (3.8)	18 (4.0)	

* comparing the 2 following categories: within the network (n = 87) and outside the network (n = 105)

** categories with small values were combined to abide by the rules of used statistical tests.

Interviewed participants were generally satisfied and noticed the introduction of new services such as dental services, nutrition and psychosocial support. In addition, certain medications became more available and the number of health workers was reported as having increased.

‘*Yes, it has changed, there has been improvement from 2016 onwards, such as an increase in medical staff and an increase in medicines, and there have been mobile teams at home, such as vaccination teams’* (Female- Quorqeena).

Participants suggested increasing the numbers of referral vehicles, opening psychiatric clinics, having more medications at PHCCs, and increasing the financial support of health facilities in order to serve more community members and increase the number of services.

*‘There are clinics such as [major surgeries] obstetrics, orthopedics, neurology, and advanced imaging that do not exist at all, and if any, they are in areas far from Quorqeena’* (Female- Quorqeena).

## Discussion

The Harim network evolved in response to a particular set of challenges and opportunities, including high population health needs (including for chronic care), a governance gap in a contested and shifting political landscape, and residual capacity in the health system but which was fragmented, including through provision of uncoordinated external support. The approach, guided by WHO but supported by a number of development partners, has interest for other settings of protracted emergency with similar needs. The focus has been on standardising inputs, processes and reporting and improving coordination between included NGOs as a seed for post-recovery system strengthening.

The ‘integrated network’ model still faces challenges for integration (as some services, such as immunization, community care, mental health are not integrated) and networking (as there remain gaps in connecting services and resources across facilities in the catchment area served by the network, as all facilities within the geographical setting do not participate in the network), but nevertheless represents an ambitious approach to working in a complex and dynamic emergency with high needs and high expectations.

Judged in relation to its original goals, which focused on comprehensive, coordinated services, equitable access and efficient service delivery, our study findings suggest that gains have been made in all three areas through the network, although assessing attribution is challenging, given the likely spread of training and tools to other facilities and the complex environment, with multiple support (funding) streams for each of the NGOs and facilities. In relation to comprehensive and coordinated services, our findings suggest that the network has achieved significant gains in this domain, in particular in relation to improvements in knowledge by facilities of each other’s services, systems for referring patients, and sharing of information with the community about service availability.

Other mechanisms of change here have included better coordination of partners and standardisation of protocols, as well as establishing a clear service package and its needed inputs at different facility types. At the same time, there are areas of weakness which have been highlighted, including continuing overlap in services and coverage areas, and lack of integration with the package of community health and vertical disease programmes (tuberculosis), though this is an area which may be addressed in future expansion.

In relation to equitable access, within the network (and indeed more broadly for NGO services) users should not be charged for services, and our patient survey suggests gains in terms of reducing financial barriers for both pregnant women and people living with NCDs. There are also encouraging results for cultural, psychological and security barriers, though these are more differentiated by user group. Our findings also indicate possible efficiency gains, although these would require more analysis.

This network model is unusual and it is likely that some very particular characteristics of the Syrian situation (such as the high residual infrastructure from a more settled era, as well as the availability of local NGOs able to provide the services) made this network approach possible. This is important to bear in mind when considering the lessons for other areas facing complex emergencies [15, 16].

Considering this model in relation to the framework for integrated people-centred service [17], it is clear that the Harim model prioritised certain elements within that framework – especially reorienting the model of care and coordinating services within and across sectors. This may be appropriate within the humanitarian context, but given the protracted emergency context, strengthening governance and accountability should be given priority too.

Reflecting on resilience capacities for health systems in fragile and shock-prone settings [18], the Harim network has invested in pathways to increase the availability, capacity and motivation of human resources, although considerable challenges still remain for this domain, and also of physical and financial resources, while also growing the networks and collaboration capacities. However, areas of relative weakness have emerged in relation to learning across the network, emergency planning and effective use of information systems. Over time the network has demonstrated absorptive, adaptive and even, more recently, transformative capacities [19] – for example, making changes to the EHSP, adopting a broader governance platform recommendation, and introducing common reporting tools.

Some conducive conditions may be derived from this experience, whose presence might support similar approaches elsewhere and which indicate areas for potential investment. These include a degree of at least temporary stability in the area and the presence of an active coordinating body, such as the Health Cluster, which has been able to manage to an extent fund flows and also procurement of supplies via donors. It has also been important to have a central coordinating body with credibility to establish technical norms and systems (the WHO Gaziantep office, in this case). Some reporting platforms were also functional – the 4Ws and HeRAMS reporting system by NGOs, for example - even though a more comprehensive system which includes verification was not in place. The presence of de facto local heath governance by the health directorates was also supportive, as was the presence of local NGOs with the ability to operate services, and a system for procuring supplies across the border. There have also been interested development partners able to provide at least minimal financial support, and a reserve of trained staff in the region (although that has been at risk of depletion, with movements and a fall in training).

A number of key areas arise for consideration to strengthen the functioning of the network in the future and as it expands in the region, including working with decentralised de facto health authorities to increase integration but also to build structures with a legacy post-emergency. Stronger governance would also potentially enable the network to manage challenges such as HR movement across facilities, the need for streamlined procurement and information systems, and stronger emergency planning and integration of vertical service provision.

## Conclusion

The article adds to a currently limited published literature on use of networks in fragile and shock prone settings. In it we examine an innovative networked service delivery model which was piloted and scaled up in northwest Syria, led by the WHO Field Presence in Gaziantep. By defining an EHSP and providing support to its implementation, including quality improvement, training, supply chain support and monitoring, the network has contributed to improving the comprehensiveness and coordination of services in its area and likely their equity and efficiency. However, many challenges remain, including for sustainability and scale up in a dynamic region, which continues to be affected by insecurity and relies on cross-border supplies and funding. It is nevertheless important to share lessons from this model, which has demonstrated some resilience capacities.

## Electronic supplementary material

Below is the link to the electronic supplementary material.

Supplementary Material 1

## Data Availability

The datasets used and/or analysed during the current study are available from the corresponding author on reasonable request.

## Footnotes

1 Health clusters are humanitarian coordination mechanisms. There are currently 32 worldwide. https://www.humanitarianresponse.info/en/coordination/clusters/health.

## Abbreviations

BP: Blood pressure
CHWs: Community health workers
CLD: Casual loop diagrams
DHIS2: Digital Health Information System 2
DM: Diabetes Mellitus
ECHO: European Civil Protection and Humanitarian Aid Operations
EHSP: Essential Health Services package
EmONC: Emergency Obstetrics and Newborn Care
ENT: Ear, nose and throat
FCDO: Foreign, Commonwealth & Development Office
GI: Gastrointestinal
GMB: Group model building
HeRAMS: The Health Resources and Services Availability Monitoring System
HTN: Hypertension
IDPs: Internally displaced persons
iNGOs: international non-governmental organisations
IPs: Implementing partners
KIIs: Key-informant interviews
KIs: Key-informants
MAM: Moderate Acute Malnutrition
mhGAP: Mental Health Gap Action Programme
MS: Microsoft
NCDs: Non-communicable diseases
NGOs: Non-governmental organisations
NWS: Northwest Syria
OFDA: Office of U.S. Foreign Disaster Assistance
PHCCs: Primary healthcare centers
SAM: Severe Acute Malnutrition
UNSCR: UN Security Council Resolution
WHO: World Health Organisation
WHO PEN: WHO package of essential noncommunicable
