# Supplementary material for: Evolution and lessons from an integrated service delivery network in North West Syria
Source: Confl Health. 2023 Mar 24;17:12. doi: 10.1186/s13031-023-00510-3 (PMC10037359; doi:10.1186/s13031-023-00510-3)
Supplement: Supplementary file 1 — Supplementary Material 1 [file 13031_2023_510_MOESM1_ESM.docx]

Appendix 1**: Description of activities carried out in each GMB workshop**

| **Activity title** | **Description of activity, by workshop** | |
| --- | --- | --- |
|  | **Workshop 1: Donors and WHO representatives** | **Workshop 2: Network staff and health professionals** |
| Reference modes: elicitation of perceptions on key trends over time | Focused on period from 2014-15 (pre-network) onward, two groups drawing perceptions on trends relating to: security, number of NGOs involved in network over time (and facilities with specialist profiles), funding availability of the network, coordination of services, level of human resources available in the region, area level service coverage and equity thereof, quality of care, data reporting | Focused on period from 2014-15 (pre-network) onward, groups focused on drawing perceptions on trends relating to: availability of human resources, quality of services delivered, total number of health facilities, number of health managers and service coordinators |
| Variable elicitation | Participants are asked to reflect on the trends above and identify key variables affecting how the network performs; a long list of variables of relevance to why the network was created and its functions, including influences on performance was then developed | |
| Causal loop diagram development | Participants focused on developing a causal loop model reflecting on two main issues: 1) the situation that prompted the emergence of the network and 2) the current challenges affecting network function. In relation to the latter, participants focused on diverse points. Donors and WHO representatives focused on distal and macro-level challenges to the network, while health professional and coordinators focused on more proximal issues. | |

Appendix 2**: Sample size overview for cross-sectional survey**

| **Patient group** | **Outcome** | **Assumptions** | **Sample size** | **Participants recruited** |
| --- | --- | --- | --- | --- |
| Adult pregnant women, at least in 2^nd^ trimester | Receiving all three service elements during consultations: blood pressure measurement, folic acid supplementation, counselling on pregnancy danger signs | Assuming 20% difference in proportion of women receiving all three services between facilities within the network (60%) and those outside (40%), 80% power, 5% significance | 208 women, 104 per group | 233 |
| Adult patients reporting with hypertension and/or diabetes | Receiving one service element during consultations: assessment of blood pressure or blood glucose (or both) | Assuming 20% difference in proportion of persons receiving these services between facilities within the network (80%) and those outside (60%), 80% power, 5% significance | 178 persons, 89 per group | 214 |
| **Total sample** | | | **386** | **447** |

Appendix 3**.** Table 1: Characteristics of the sample of people living in Idleb (N=447)

| **Variables** | **Pregnant women**  **(n=233) *** | **People living with NCD (n=214)** | **Total**  **(N=447)** |
| --- | --- | --- | --- |
| **Age in years (mean; SD)** | 27.8 (6.0) | 52.7 (9.4) | 39.6 (14.7) |
| **Gender (n, %)** |  |  |  |
| Female | 233 (100) | 132 (61.7) | 365 (81.7) |
| Male | 0 (0.0) | 79 (36.9) | 79 (17.7) |
| Prefer not to mention | 0 (0.0) | 3 (1.4) | 3 (0.7) |
| **Marital status (n, %)** |  |  |  |
| Married | 230 (98.7) | 169 (79.0) | 399 (89.3) |
| Separated/Divorced | 1 (0.4) | 3 (1.4) | 4 (0.9) |
| Widowed | 2 (0.9) | 42 (19.6) | 44 (9.8) |
| Single | 0 (0.0) | 0 (0.0) | 0 (0.0) |
| **Educational level (n, %)** |  |  |  |
| Primary school or less | 130 (60.7) | 66 (28.3) | 196 (43.8) |
| Secondary school | 61 (28.5) | 111 (47.6) | 172 (38.5) |
| High school | 17 (7.9) | 38 (16.3) | 55 (12.3) |
| University | 6 (2.8) | 18 (7.7) | 24 (5.4) |
| **Employment status (n, %)** |  |  |  |
| Employed | 20 (8.6) | 14 (6.5) | 34 (7.6) |
| Unemployed | 212 (91.0) | 179 (83.6) | 391 (87.5) |
| Retired | 1 (0.4) | 21 (9.8) | 22 (4.9) |
| **Residency status (n, %)** |  |  |  |
| Internally Displaced Person (IDP) | 176 (75.5) | 173 (80.8) | 349 (78.1) |
| Resident (host communities) | 57 (24.5) | 41 (19.2) | 98 (21.9) |
| **Residence type (n, %)** |  |  |  |
| Tent | 101 (43.3) | 54 (25.2) | 155 (34.7) |
| A room within another household | 24 (10.3) | 5 (2.3) | 29 (6.5) |
| House or apartment | 108 (46.4) | 155 (72.4) | 263 (58.8) |
| **Crowding index (mean ± SD)** | 3.54 (1.79) | 3.16 (1.57) | 3.36 (1.70) |
| **Facility (n, %)** |  |  |  |
| **Hospital outpatient clinic 1 – within Harim Network (HN)** | 29 (12.4) | 30 (14.0) | 59 (13.2) |
| Hospital outpatient clinic 2 – outside HN | 28 (12.0) | 30 (14.0) | 58 (13.0) |
| **PHCC 1 – within HN** | 29 (12.4) | 30 (14.0) | 59 (13.2) |
| PHCC 2 – outside HN | 47 (20.2) | 30 (14.0) | 77 (17.2) |
| **PHCC 3 – within HN** | 29 (12.4) | 30 (14.0) | 59 (13.2) |
| PHCC 4 – outside HN | 30 (12.9) | 25 (11.7) | 55 (12.3) |
| MC1 – outside HN | 24 (10.3) | 19 (8.9) | 43 (9.6) |
| MC2 – outside HN | 17 (7.3) | 20 (9.3) | 37 (8.3) |

* including 18 pregnant women living with hypertension and/or diabetes

Appendix 3**.** Table 2: Access to care among people living in Idleb, by facility type/affiliation to the network (N=447)

| **Variables** | **Within the network (n=177)** | **Outside the network (n=190)** | **Mobile Clinics (n=80)** | **Total**  **(N=447)** | **p-value*** |
| --- | --- | --- | --- | --- | --- |
| **Security and movement barriers (n,%)** | | | | | **0.023** |
| Never | 78 (44.1) | 66 (34.7) | 24 (30.0) | 168 (37.6) |  |
| Rarely | 48 (27.1) | 46 (24.2) | 25 (31.3) | 119 (26.6) |  |
| Sometimes | 28 (15.8) | 37 (19.5) | 22 (27.5) | 87 (19.5) |  |
| Often | 20 (11.3) | 26 (13.7) | 5 (6.3) | 51 (11.4) |  |
| Every time | 3 (1.7) | 15 (7.9) | 4 (5.0) | 22 (4.9) |  |
| **Physical/geographical barrier (n,%)** | | |  |  | **0.025** |
| Never | 44 (24.9) | 33 (17.4) | 10 (12.5) | 87 (19.5) |  |
| Rarely | 40 (22.6) | 27 (14.2) | 22 (27.5) | 89 (19.9) |  |
| Sometimes | 36 (20.3) | 42 (22.1) | 18 (22.5) | 96 (21.5) |  |
| Often | 33 (18.6) | 55 (28.9) | 21 (26.3) | 109 (24.4) |  |
| Every time | 24 (13.6) | 33 (17.4) | 9 (11.3) | 66 (14.8) |  |
| **Cultural barrier (e.g. gender sensitivity, cultural differences between patients and providers)** | | |  |  | **0.038**** |
| Never | 106 (59.9) | 96 (50.5) | 39 (48.8) | 241 (53.9) |  |
| Rarely | 40 (22.6) | 39 (20.5) | 20 (25.0) | 99 (22.1) |  |
| Sometimes | 19 (10.7) | 26 (13.7) | 14 (17.5) | 59 (13.2) |  |
| Often | *11 (6.2)* | *25 (13.2)* | *7 (8.8)* | *43 (9.6)* |  |
| Every time | *1 (0.6)* | *4 (2.1)* | *0 (0.0)* | *5 (1.1)* |  |
| **Health literacy-related barrier (e.g. not knowing when to seek healthcare support)** | | |  |  | **<0.001** |
| Never | 10 (5.6) | 34 (17.9) | 9 (11.3) | 53 (11.9) |  |
| Rarely | 41 (23.2) | 30 (15.8) | 18 (22.5) | 89 (19.9) |  |
| Sometimes | 68 (38.4) | 58 (30.5) | 37 (46.3) | 163 (36.5) |  |
| Often | 40 (22.6) | 57 (30.0) | 11 (13.8) | 108 (24.2) |  |
| Every time | 18 (10.2) | 11 (5.8) | 5 (6.3) | 34 (7.6) |  |
| **Psychological barrier (e.g. fear to get diagnosed with more diseases)** | | |  |  | 0.670 |
| Never | 58 (32.8) | 63 (33.2) | 37 (46.3) | 158 (35.3) |  |
| Rarely | 45 (25.4) | 40 (21.1) | 18 (22.5) | 103 (23.0) |  |
| Sometimes | 41 (23.2) | 41 (21.6) | 16 (20.0) | 98 (21.9) |  |
| Often | 23 (13.0) | 34 (17.9) | 8 (10.0) | 65 (14.5) |  |
| Every time | 10 (5.6) | 12 (6.3) | 1 (1.3) | 23 (5.1) |  |
| **Financial barrier (including direct and indirect costs)** | | | | | **<0.001** |
| Never | 47 (26.6) | 25 (13.2) | 9 (11.3) | 81 (18.1) |  |
| Rarely | 32 (18.1) | 21 (11.1) | 7 (8.8) | 60 (13.4) |  |
| Sometimes | 33 (18.6) | 47 (24.7) | 14 (17.5) | 94 (21.0) |  |
| Often | 24 (13.6) | 56 (29.5) | 34 (42.5) | 114 (25.5) |  |
| Every time | 41 (23.2) | 41 (21.6) | 16 (20.0) | 98 (21.9) |  |

- * comparing the 2 following categories: within the network (n=87) and outside the network (n=105)
- ** categories with small values were combined to abide by the rules of used statistical tests.

Appendix 3**.Table 3a:** Experiences with service delivery and satisfaction levels among people living with NCD in Idleb, by facility type/affiliation to the network (N=214)

| **Variables** | **Within the network (n=90)** | **Outside the network (n=85)** | **Mobile Clinics (n=39)** | **Total**  **(N=214)** | **p-value*** |
| --- | --- | --- | --- | --- | --- |
| First contact – going to the facility as a first option | 79 (87.8) | 62 (72.9) | 32 (82.1) | 173 (80.8) | **0.022** |
| **Confidence to be seen on the same day** | | | | | 0.999** |
| Strongly disagree | *0 (0.0)* | *11 (12.9)* | *0 (0.0)* | *11 (5.1)* |  |
| Disagree | *12 (13.3)* | *0 (0.0)* | *3 (7.7)* | *15 (7.0)* |  |
| Neutral | 11 (12.2) | 11 (12.9) | 5 (12.8) | 27 (12.6) |  |
| Agree | 63 (70.0) | 59 (69.4) | 30 (76.9) | 152 (71.0) |  |
| Strongly agree | 4 (4.4) | 4 (4.7) | 1 (2.6) | 9 (4.2) |  |
| **Doctors/nurses knowing what problems important to the beneficiary** | | | | | **0.024**** |
| Strongly disagree | *0 (0.0)* | *2 (2.4)* | *0 (0.0)* | *2 (0.9)* |  |
| Disagree | *9 (10.0)* | *1 (1.2)* | *10 (25.6)* | *20 (9.3)* |  |
| Neutral | 12 (13.3) | 22 (25.9) | 3 (7.7) | 37 (17.3) |  |
| Agree | 65 (72.2) | 51 (60.0) | 25 (64.1) | 141 (65.9) |  |
| Strongly agree | 4 (4.4) | 9 (10.6) | 1 (2.6) | 14 (6.5) |  |
| **Doctors/nurses asking about referral visits to other specialists/services** | | | | | **0.040**** |
| Strongly disagree | *0 (0.0)* | *7 (8.2)* | *0 (0.0)* | *7 (3.3)* |  |
| Disagree | *13 (14.4)* | *2 (2.4)* | *5 (12.8)* | *20 (9.3)* |  |
| Neutral | *0 (0.0)* | *12 (14.1)* | *1 (2.6)* | *13 (6.1)* |  |
| Agree | 69 (76.7) | 50 (58.8) | 32 (82.1) | 151 (70.6) |  |
| Strongly agree | 8 (8.9) | 14 (16.5) | 1 (2.6) | 23 (10.7) |  |
| **Patient satisfaction scores** | | | | | |
| **Satisfaction regarding health staff during consultations** | | | | | |
| 1.Provider skills | 4.29 (0.67) | 3.84 (0.77) | 3.72 (0.99) | 4.00 (0.81) | **<0.001** |
| 2.Being spoken to respectfully | 4.30 (0.57) | 4.05 (0.80) | 4.08 (1.04) | 4.16 (0.77) | 0.018 |
| 3.Privacy during consultation | 4.22 (0.70) | 4.11 (0.76) | 3.92 (1.33) | 4.12 (0.87) | 0.292 |
| 4.Explanations about treatment options and alternatives | 3.62 (0.73) | 3.69 (0.82) | 3.44 (0.85) | 3.62 (0.79) | 0.539 |
| 5.Time availability for questions and clarifications | 3.51 (0.78) | 3.67 (0.82) | 3.77 (0.90) | 3.62 (0.82) | 0.190 |
| 6.Clarity of explanations during consultation | 3.68 (0.70) | 3.87 (0.81) | 3.62 (0.91) | 3.74 (0.79) | 0.094 |
| 7.Involvement in decision making about your health and treatment | 3.42 (0.72) | 3.74 (0.74) | 2.92 (1.06) | 3.46 (0.85) | **0.004** |
| 8.Confidentiality of your personal information | 4.32 (0.52) | 4.29 (0.72) | 4.62 (0.85) | 4.36 (0.68) | 0.768 |
| 9. Availability of a private space for examination | 4.09 (0.59) | 3.99 (0.94) | 3.38 (1.07) | 3.92 (0.88) | 0.403 |
| 10. Cleanliness of the space for examination | 4.20 (0.52) | 4.33 (0.73) | 4.36 (0.84) | 4.28 (0.67) | 0.182 |
| **Satisfaction regarding facilities** | | | | | |
| 11.Waiting time for appointment scheduling | 3.89 (0.76) | 3.29 (1.01) | 3.44 (0.94) | 3.57 (0.94) | **<0.001** |
| 12.Waiting time in facility (for receiving services) | 3.67(0.64) | 3.18 (0.98) | 3.44 (0.99) | 3.43 (0.88) | **<0.001** |
| 13.Waiting space (availability, crowdedness) | 3.64 (0.71) | 3.32 (0.86) | 3.18 (1.29) | 3.43 (0.91) | **0.007** |
| 14. Availability of equipment | 3.53 (0.78) | 3.82 (0.94) | 3.97 (0.90) | 3.73 (0.88) | **0.028** |
| 15. Condition and functionality of equipment | 3.58 (0.85) | 3.95 (0.90) | 4.05 (0.99) | 3.81 (0.91) | **0.005** |
| 16. Availability of medication | 2.66 (0.80) | 2.53 (0.97) | 1.95 (0.99) | 2.48 (0.94) | 0.347 |
| 17.Facility cleanliness (including toilets) | 4.00 (0.56) | 3.95 (0.75) | 4.00 (0.97) | 3.98 (0.72) | 0.642 |
| 18.Staff friendliness and greetings | 4.18 (0.53) | 4.06 (0.85) | 4.10 (1.19) | 4.12 (0.81) | 0.272 |
| **Satisfaction regarding community health workers** | | | | | |
| 19. Community health worker skills | 3.68 (0.79) | 4.04 (0.76) | 3.59 (0.85) | 3.83 (0.81) | **0.008** |
| 20. Privacy and confidentiality | 3.95 (0.72) | 4.22 (0.85) | 4.00 (1.12) | 4.09 (0.88) | **0.039** |
| 21. Explanations about health issues | 3.37 (0.71) | 4.01 (0.84) | 3.31 (0.89) | 3.65 (0.87) | **<0.001** |
| 22. Advice on when to seek care from facility | 3.63 (0.82) | 4.24 (0.83) | 3.54 (1.05) | 3.89 (0.93) | **<0.001** |

* comparing the 2 following categories: within the network (n=90) and outside the network (n=85)

** categories with small values were combined to abide by the rules of used statistical tests.

Appendix 3**.Table 3b:** Experiences with service delivery and satisfaction levels among pregnant women living with NCD in Idleb, by facility type/affiliation to the network (N=233)

| **Variables** | **Within the network (n=87)** | **Outside the network (n=105)** | **Mobile Clinics (n=41)** | **Total**  **(N=233)** | **p-value*** |
| --- | --- | --- | --- | --- | --- |
| **First contact – going to the facility as a first option** | 82 (94.3) | 101 (96.2) | 39 (95.1) | 222 (95.3) | 0.734 |
| **Confidence to be seen on the same day** | | | | | 0.316** |
| Strongly disagree | *0 (0.0)* | *1 (1.0)* | *0 (0.0)* | *1 (0.4)* |  |
| Disagree | *14 (16.1)* | *8 (7.6)* | *2 (4.9)* | *24 (10.3)* |  |
| Neutral | 8 (9.2) | 8 (7.6) | 3 (7.3) | 19 (8.2) |  |
| Agree | 60 (69.0) | 84 (80.0) | 27 (65.9) | 171 (73.4) |  |
| Strongly agree | 5 (5.7) | 4 (3.8) | 9 (22.0) | 18 (7.7) |  |
| **Doctors/nurses knowing what problems important to the beneficiary** | | | | | 0.289 |
| Strongly disagree | *0 (0.0)* | *0 (0.0)* | *0 (0.0)* | *0 (0.0)* |  |
| Disagree | 13 (14.9) | 10 (9.5) | 2 (4.9) | 25 (10.7) |  |
| Neutral | 10 (11.5) | 9 (8.6) | 4 (9.8) | 23 (9.9) |  |
| Agree | 61 (70.1) | 77 (73.3) | 29 (70.7) | 167 (71.7) |  |
| Strongly agree | 3 (3.4) | 9 (8.6) | 6 (14.6) | 18 (7.7) |  |
| **Doctors/nurses asking about referral visits to other specialists/services** | | | | | **< 0.001** |
| Strongly disagree | *0 (0.0)* | *0 (0.0)* | *0 (0.0)* | *0 (0.0)* |  |
| Disagree | 6 (6.9) | 27 (25.7) | 1 (2.4) | 34 (14.6) |  |
| Neutral | 10 (11.5) | 3 (2.9) | 2 (4.9) | 15 (6.4) |  |
| Agree | 68 (78.2) | 64 (61.0) | 30 (73.2) | 162 (69.5) |  |
| Strongly agree | 3 (3.4) | 11 (10.5) | 8 (19.5) | 22 (9.4) |  |
| **Patient satisfaction scores** | | | | | |
| **Satisfaction regarding health staff during consultations** | | | | | |
| 1.Provider skills | 4.11 (0.77) | 4.36 (0.62) | 3.73 (0.77) | 4.16 (0.74) | **0.017** |
| 2.Being spoken to respectfully | 4.25 (0.78) | 4.33 (0.77) | 4.15 (0.85) | 4.27 (0.79) | 0.474 |
| 3.Privacy during consultation | 4.57 (0.71) | 4.14 (0.89) | 4.37 (0.83) | 4.34 (0.84) | **<0.001** |
| 4.Explanations about treatment options and alternatives | 3.64 (0.96) | 3.70 (0.89) | 3.41 (0.77) | 3.63 (0.90) | 0.701 |
| 5.Time availability for questions and clarifications | 3.64 (0.96) | 3.51 (0.92) | 3.51 (0.67) | 3.56 (0.90) | 0.344 |
| 6.Clarity of explanations during consultation | 3.77 (1.01) | 3.72 (0.92) | 3.68 (0.99) | 3.73 (0.96) | 0.741 |
| 7.Involvement in decision making about your health and treatment | 3.41 (1.13) | 3.65 (0.99) | 3.34 (0.86) | 3.51 (1.03) | 0.128 |
| 8.Confidentiality of your personal information | 4.68 (0.72) | 4.43 (0.76) | 4.51 (0.78) | 4.54 (0.75) | **0.021** |
| 9. Availability of a private space for examination | 4.57 (0.76) | 4.52 (0.69) | 3.51 (1.33) | 4.36 (0.94) | 0.631 |
| 10. Cleanliness of the space for examination | 4.78 (0.58) | 4.32 (0.79) | 4.02 (1.04) | 4.44 (0.82) | **<0.001** |
| **Satisfaction regarding facilities** | | | | | |
| 11.Waiting time for appointment scheduling | 3.75 (1.09) | 3.50 (0.93) | 3.51 (0.68) | 3.60 (0.96) | 0.099 |
| 12.Waiting time in facility (for receiving services) | 3.54 (1.04) | 3.31 (0.86) | 3.51 (0.84) | 3.43 (0.93) | 0.101 |
| 13.Waiting space (availability, crowdedness) | 3.84 (0.97) | 3.47 (1.00) | 3.22 (0.99) | 3.56 (1.01) | **0.010** |
| 14. Availability of equipment | 3.86 (0.85) | 3.92 (0.82) | 4.10 (0.83) | 3.93 (0.83) | 0.610 |
| 15. Condition and functionality of equipment | 3.75 (0.99) | 3.88 (0.86) | 4.02 (0.76) | 3.85 (0.90) | 0.342 |
| 16. Availability of medication | 2.92 (1.04) | 2.71 (1.05) | 3.15 (1.08) | 2.87 (1.06) | 0.177 |
| 17.Facility cleanliness (including toilets) | 4.43 (0.66) | 4.02 (0.85) | 3.59 (1.14) | 4.09 (0.89) | **<0.001** |
| 18.Staff friendliness and greetings | 4.25 (0.77) | 4.30 (0.80) | 4.29 (0.81) | 4.28 (0.78) | 0.709 |
| **Satisfaction regarding community health workers** | | | | | |
| 19. Community health worker skills | 3.88 (0.96) | 4.09 (0.65) | 4.05 (0.86) | 4.02 (0.79) | 0.146 |
| 20. Privacy and confidentiality | 4.14 (1.07) | 4.04 (0.81) | 4.34 (0.85) | 4.13 (0.90) | 0.536 |
| 21. Explanations about health issues | 3.83 (1.06) | 4.08 (0.85) | 3.83 (0.92) | 3.96 (0.93) | 0.105 |
| 22. Advice on when to seek care from facility | 3.90 (1.10) | 4.10 (0.81) | 4.20 (0.95) | 4.06 (0.93) | 0.210 |

* comparing the 2 following categories: within the network (n=87) and outside the network (n=105)

** categories with small values were combined to abide by the rules of used statistical tests.
